# Specificity and Sensitivity of Circulating Tumor HPV-DNA in Patients with HPV-Positive Oropharyngeal Squamous Cell Carcinoma

**DOI:** 10.3390/diagnostics16142169

**Published:** 2026-07-11

**Authors:** Jakob Myllerup Jensen, Simone Kloch Bendtsen, Kathrine Kronberg Jakobsen, Benedicte Bitsch Lauritzen, Katalin Kiss, Maria Rossing, Jeppe Friborg, Christian Grønhøj, Christian von Buchwald

**Affiliations:** 1 Department of Otorhinolaryngology, Head and Neck Surgery and Audiology, Copenhagen University Hospital, Rigshospitalet, 2100 Copenhagen, Denmark; 2 Department of Pathology, Copenhagen University Hospital, Rigshospitalet, 2100 Copenhagen, Denmark; 3 MDxCORE, Department of Clinical Biochemistry, Copenhagen University Hospital, Rigshospitalet, 2100 Copenhagen, Denmark; 4 Department of Clinical Medicine, Faculty of Health and Medical Sciences, University of Copenhagen, 2200 Copenhagen, Denmark; 5 Department of Oncology, Copenhagen University Hospital, Rigshospitalet, 2100 Copenhagen, Denmark

**Keywords:** oropharyngeal squamous cell carcinoma, OPSCC, HPV, ctHPV-DNA, biomarker

## Abstract

The global incidence of oropharyngeal squamous cell carcinoma (OPSCC) is increasing, mainly due to a rise in human papillomavirus-positive (HPV+) cases. In Denmark, this group now constitutes more than 70% of newly diagnosed patients with OPSCC, making HPV+ OPSCC an increasing health issue. Approximately 10–15% of patients experience recurrence after treatment, and 30% die within five years of diagnosis. Detecting recurrence can be challenging, as it relies on either visible findings during clinical examination or symptoms reported by the patient. However, recent studies have shown that circulating tumor HPV-DNA (ctHPV-DNA) is a promising biomarker for detecting recurrence. The aim of the study presented in this protocol is to investigate whether prospective and systematic ctHPV-DNA testing can improve the standard follow-up program by detecting recurrence earlier than, or simultaneously with, clinical or radiological examination. This study is a prospective diagnostic accuracy study aiming to include 200 newly diagnosed patients with HPV+ OPSCC. Patients will be followed systematically with blood samples at baseline before treatment initiation and at every standard follow-up visit after treatment completion for up to three years. If a sample after treatment completion is positive for ctHPV-DNA, the patient will be referred for clinical ENT examination and a PET/CT scan to assess if the patient has detectable recurrence. This study will provide novel knowledge of the sensitivity and specificity of ctHPV-DNA testing for detecting recurrence in patients with HPV+ OPSCC, based on a large, prospectively and systematically followed cohort in which positive ctHPV-DNA findings will be acted upon clinically.

## 1. Introduction

### 1.1. Background

Approximately 1600 patients are diagnosed with head and neck cancer (HNC) in Denmark annually, and the incidence is increasing [1]. Oropharyngeal squamous cell carcinoma (OPSCC) constitutes one of the largest subgroups of HNC and the fastest-increasing incidence [1]. This increase is primarily driven by the rising incidence of human papillomavirus-positive (HPV+) OPSCC [2,3,4,5,6,7], which now accounts for up to 70% of all OPSCC cases in Denmark [2]. Compared with HPV-negative (HPV−) OPSCC patients, patients with HPV+ OPSCC have a distinct clinical profile, and their tumors differ both molecularly and histopathologically. HPV is a group of double-stranded DNA viruses [8], and several different high-risk genotypes exist. The most prevalent genotypes in OPSCC in Denmark are HPV16, HPV33, HPV35, HPV18, and HPV45 [2]. HPV+ patients are typically younger, have fewer comorbidities, and have a better prognosis than HPV-patients [9,10]. Despite this, 10–15% of patients with HPV+ OPSCC experience recurrence despite successful treatment, and 30% die within 5 years of diagnosis [11]. In Denmark, all patients, regardless of HPV status, treatment modality, or disease stage, follow the same standardized clinical follow-up program to facilitate early detection of recurrence. However, detecting recurrence remains challenging, as it relies on either visible findings during clinical examination or symptoms reported by the patient.

A previous study from our department demonstrated that circulating tumor HPV-DNA (ctHPV-DNA) was detectable at the time of diagnosis of HPV+ OPSCC with a sensitivity of 97%, and that ctHPV-DNA levels decreased after treatment [12]. In patients whose primary treatment was successful, ctHPV-DNA levels dropped below the limit of detection, whereas ctHPV-DNA could still be detected in the blood of patients with residual disease [12]. Reappearing ctHPV-DNA was detected in patients with recurrence up to four months before recurrent disease was diagnosed clinically [12]. These findings suggest that ctHPV-DNA testing may detect recurrence earlier than conventional clinical examination, thereby enabling earlier diagnosis and potentially earlier intervention. However, these findings require validation in a prospective setting.

### 1.2. Objectives

The aim of this study is to investigate whether prospective and systematic ctHPV-DNA testing can improve the standard follow-up program for patients with HPV+ OPSCC by detecting recurrence earlier than, or simultaneously with, conventional clinical or radiological assessment.

## 2. Methods: Patient and Public Involvement, Trial Design

### 2.1. Patient and Public Involvement

The project was presented to the Danish Head and Neck Cancer Patient Association and received very positive feedback. The association has also contributed to drafting the written patient information to ensure clarity and readability.

To assess how the ctHPV-DNA test and its results influence patients’ quality of life, patients will be asked to complete the Danish version of the Fear of Cancer Recurrence Questionnaire (short form) [13,14] after receiving the result of each blood sample.

### 2.2. Trial Design

This study is a prospective diagnostic accuracy study investigating the clinical value of adding ctHPV-DNA testing to the existing standard follow-up program for patients treated for HPV+ OPSCC.

## 3. Methods: Participants, Interventions, and Outcomes

### 3.1. Trial Settings

The study will be conducted at the Department of Otorhinolaryngology, Head and Neck Surgery and Audiology, at Copenhagen University Hospital—Rigshospitalet, Copenhagen, Denmark. Patients are identified through the diagnostic Cancer Fast-Track pathway. Here, all patients from Eastern Denmark are examined and diagnosed before treatment initiation.

### 3.2. Eligibility Criteria

The study will include all newly diagnosed patients with HPV+ OPSCC in Eastern Denmark.

Inclusion criteria are as follows:

• Patients with newly diagnosed OPSCC positive for HPV16, HPV18, HPV31, HPV33, HPV35, HPV45, HPV51, or HPV58.
• Age ≥ 18 years.
• Informed consent.

Exclusion criteria are as follows:

• Inability to speak or understand Danish.
• Patients receiving palliative treatment.

### 3.3. Intervention and Comparator

This is a non-randomized study, including all patients diagnosed with HPV+ OPSCC. Samples will be collected from included patients at the time of diagnosis (baseline), at the follow-up visit two months after completion of treatment, and every six months thereafter for a minimum of two years and up to three years. Each sample will be analyzed for the presence of ctHPV-DNA corresponding to the HPV genotype identified in the patient’s primary tumor. If a sample is positive after completion of treatment, the patient will be referred for a clinical examination at the Department of Otolaryngology, Head and Neck Surgery and Audiology, Copenhagen University Hospital—Rigshospitalet, followed by a PET/CT scan (supplemented by MRI if a tumor is clinically visible in the head and neck region) within 12 weeks.

### 3.4. Outcomes

The primary endpoint is to evaluate the sensitivity and specificity of ctHPV-DNA for the detection of recurrence in patients with HPV+ OPSCC, using clinically or radiologically verified recurrence as the reference standard. Time frame: A minimum of two years and up to three years after completion of primary treatment. Sensitivity will be calculated among patients who develop recurrence, whereas specificity will be calculated among patients who remain recurrence-free.

Secondary endpoints are defined as follows:

1. Time to recurrence detected by ctHPV-DNA.
2. Time to recurrence detected by clinical and/or radiological examination.
3. Number of referrals to Cancer Fast-Track examinations based on a positive ctHPV-DNA test result without evidence of recurrence.
4. Number of PET/CT scans performed because of a positive ctHPV-DNA test result without evidence of recurrence.

Quality of life (QoL), assessed using the Danish version of the Fear of Cancer Recurrence Questionnaire (short form), will be completed by patients after receiving the result of each blood sample.

### 3.5. Harms

In general, blood sampling is considered a minimally invasive and simple procedure. When blood is drawn, transient pain at the venepuncture site and minor bruising may occur. This will pass within a few days. Patients participating in this study are covered by the Danish Patient Compensation Association (Patienterstatningen) in the event of study-related injury. The information leaflets “Forsøgspersoners rettigheder i sundhedsvidenskabelige forskningsprojekter” and “Før du beslutter dig” will be included with the written participant information [15,16]. Participants experiencing any adverse events that may be related to the study will be instructed to contact the study investigators.

Every patient with a positive ctHPV-DNA sample after completion of treatment will be referred for a PET/CT scan to assess whether there is evidence of clinical recurrence. The scan is a standard diagnostic PET/CT (2-(flourine-18) fluoro-2-deoxy-D-glukose (FDG)-PET/CT). During the PET part of the scan, the radioactive sugar FDG will be injected. PET has no known side effects. The CT part of the scan is performed using intravenous iodinated contrast medium. Immediately after injection, some patients may experience a transient sensation of warmth, a metallic taste, or an urge to urinate. As with any medicinal product, iodinated contrast medium carries a risk of allergic reactions upon injection. Approx. 5% of patients will experience minor side effects, including transient nausea, itching or rashes, whereas the risk of serious adverse reactions is very low (<0.1%). If the patient has previously experienced a severe reaction to IV contrast medium, the PET/CT can be conducted without the use of IV contrast medium. Some patients may experience claustrophobia during the scan, but trained staff will always be present to provide assistance if needed. Some participants may also undergo repeated PET/CT scans due to positive ctHPV-DNA blood samples and no evidence of recurrence, resulting in additional exposure to ionizing radiation. The associated risk is considered low, and only a small number of patients are expected to undergo more than one additional PET/CT scan.

Finally, patients with positive ctHPV-DNA blood samples but without evidence of recurrence may experience increased anxiety. However, this is expected to affect only a small number of patients, which is one of the reasons why the Fear of Cancer Recurrence Questionnaire has been included in the study.

## 4. Participant Timeline

**Figure 1:**
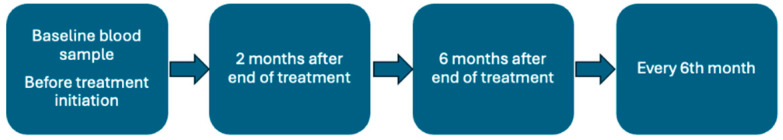
Overview of the blood sampling schedule. The first blood sample is drawn at the time of diagnosis (baseline) to assess baseline cfHPV-DNA levels before treatment initiation. The second blood sample is drawn at the first follow-up visit, two months after completion of treatment. The third blood sample is drawn six months after completion of treatment, and subsequent blood samples are collected every six months for up to three years after completion of treatment.

All included patients will be monitored with blood samples for a minimum of two years and up to three years after completion of treatment, at the same intervals as the standard clinical follow-up program (two months after completion of treatment and every six months thereafter) (Figure 1). As the median time to recurrence among patients with HPV+ OPSCC has been reported to be between 12 and 18 months, most recurrences are expected to occur within this follow-up period [17,18].

**Figure 2:**
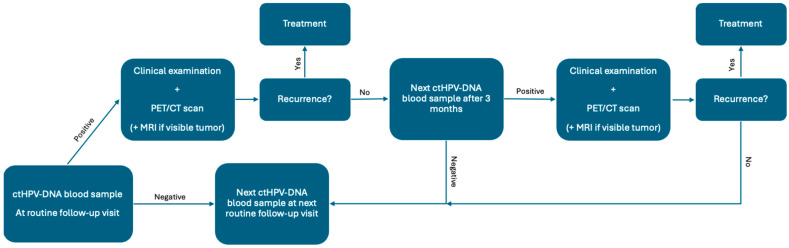
Flowchart illustrating the clinical management algorithm following ctHPV-DNA blood test results.

If a blood sample is positive at any time point after completion of treatment, the patient will be referred for a clinical examination at the Department of Otolaryngology, Head and Neck Surgery and Audiology, Copenhagen University Hospital—Rigshospitalet, followed by a PET/CT scan (supplemented by MRI if a tumor is clinically visible in the head and neck region) within 1–2 weeks. If no recurrence is detected during these examinations, blood sampling will be repeated after three months. If the additional blood sample is positive for ctHPV-DNA, a second clinical examination and PET/CT scan will be performed. If recurrence is still not detected at the additional follow-up visit, the patient will return to the standard follow-up schedule (Figure 2).

### 4.1. Sample Size

The sample size was estimated using a diagnostic accuracy study sample size calculator based on the methodology described by Akoglu et al. [19]. The calculation was based on an expected sensitivity of 95%, an expected specificity of 95%, a disease prevalence of 11%, a two-sided type I error rate of 5% (corresponding to a 95% confidence interval), and a margin of error of 10%. Based on these assumptions, a minimum of 167 patients was required. With an expected disease prevalence of 11%, this corresponds to approx. 18–19 patients with residual disease or recurrence and 148–149 patients without disease. To account for potential dropout, loss to follow-up, and non-evaluable samples, the target sample size was increased to 200 patients.

### 4.2. Recruitment

After the diagnosis of OPSCC, eligible patients are screened for eligibility before written informed consent is obtained. A recruitment letter and written participant information are sent digitally via the Danish Digital Post, inviting the patient to participate in the study. The written participant information, as well as the pamphlets “Forsøgspersoners rettigheder i et sundhedsvidenskabeligt forskningsprojekt” (“Rights of test subjects in a health scientific research project”) [15], and “Før du beslutter dig” (“Before deciding”) [16], is sent before the patient attends the first treatment consultation at the Department of Otolaryngology, Head and Neck Surgery and Audiology, Copenhagen University Hospital—Rigshospitalet. After the written participant information has been sent, a member of the research group will contact the potential participant by phone and arrange an oral presentation of the study before or after the first treatment consultation. If the potential participant does not answer the phone or respond to the invitation before the consultation, the treating physician may invite the patient to participate in the study and ask whether the patient is interested in an oral presentation of the project. If the patient wishes to participate, written informed consent is obtained.

## 5. Methods: Assignment of Interventions

### 5.1. Randomization

This study is non-randomized. All included patients will follow the same follow-up program, with blood samples collected at the same intervals as the standard clinical follow-up program for a minimum of two years and up to three years.

### 5.2. Blinding

No blinding is performed. Results of each ctHPV-DNA analysis will be communicated to the treating physician, the patient, and the study investigators.

## 6. Methods: Data Collection, Management, and Analysis

### 6.1. Data Collection Methods

The following health information will be obtained from the patients’ medical records after written informed consent has been obtained: diagnosis, date of diagnosis, anatomical location of the primary tumor, age, sex, TNM classification, UICC stage, treatment modality, dates of treatment initiation and completion, recurrence status, comorbidities, complications, previous or concurrent malignancies, performance status, smoking status, pack year history, weekly alcohol consumption, HPV and p16 status of the primary tumor and lymph nodes (including date of analysis and HPV genotype), histopathological diagnosis and morphological characteristics, imaging findings, status at the last clinical examination, date of subsequent follow-up visits, and vital status, including the date and cause of death.

Blood samples will be collected at the time of diagnosis (baseline), two months after completion of treatment, and every six months thereafter for a minimum of two years and up to three years. Each sample will be analyzed for ctHPV-DNA corresponding to the HPV genotype identified in the patient’s primary tumor. HPV genotyping on primary tumors and lymph nodes is performed using the Zytovision VisionArray HPV Chip at the Department of Pathology as part of the routine diagnostic work-up.

The blood samples are collected in Streck Cell-Free DNA BCT CE tubes. These tubes are designed to stabilize blood cells and prevent the release of genomic DNA for up to 7 days at room temperature, thereby minimizing contamination of cell-free DNA and enabling the extraction of high-quality cell-free DNA. Samples will be stored at room temperature for up to four days before processing. Approx. 8 mL plasma/patient (corresponding to two Streck Cell-Free DNA BCT CE tubes) will be isolated by centrifugation at 2250× *g* for 10 min. The isolated plasma will subsequently be centrifuged at 16,800× *g* for 10 min to remove any residual cells or cellular debris before storage at −80 °C. DNA will be extracted using the QIAamp Circulating Nucleic Acid Kit (#55114) on a QIACube instrument and eluted in 60 µL. DNA concentration and purity will be assessed using NanoDrop (Thermo Fischer Scientific, Waltham, MA, USA) or TapeStation (Agilent Technologies, Santa Clara, CA, USA). The following quality control (QC) assays will be used: QC assay 1. DNA purification efficiency will be assessed at the batch level by analyzing five healthy donor plasma samples spiked with the 191-bp CPP1 DNA spike-in before and after each batch of patient samples, instead of adding CPP1 DNA directly to the individual patient samples, allowing estimation of the mean DNA loss during extraction [20]. Batch-level controls were chosen instead of sample-level spike-in controls to avoid introducing exogenous DNA into individual clinical samples while preserving native patient DNA for future analyses. QC assay 2. Genomic DNA contamination (white blood cell contamination) will be assessed in native patient DNA as previously described [12] or using TapeStation. QC assay 3. Sample fragmentation will be assessed in native patient samples as previously described. All QC assays adhere to the guidelines published by the Danish ctDNA Research Center [12,21]. The amount of HPV-DNA in plasma and the specific HPV genotype will be assessed by our ultrasensitive droplet digital PCR (ddPCR) assay, which we have developed to assess eight high-risk HPV genotypes: HPV16, 18, 31, 33, 35, 45, 51, and 58 [12,15,16]. First, each sample will undergo a 12-cycle PCR pre-amplification containing only one HPV primer set together with the EMC7-65 primer set. Second, ddPCR analysis will be performed using the same HPV and EMC7-65 primer set (final concentration 900 nM) together with the corresponding probes (final concentration 250 nM). A sample is considered ctHPV-DNA positive when at least five HPV+ droplets are detected for the relevant HPV genotype. Samples with 5–20 HPV+ droplets, corresponding to results close to the analytical detection limit, will be confirmed using a second blood sample to minimize false-positive and false-negative results. If the second blood sample confirms HPV-DNA positivity, the sample is classified as positive. If the second blood sample is negative, the result is considered discordant, and the first blood sample is re-analyzed. The analytical limit of detection (LOD) has been internally defined as the lowest HPV-DNA concentration corresponding to five HPV+ droplets in the ddPCR analysis, corresponding to 1.9 double-stranded HPV-DNA copies/mL plasma. No formal limit of quantification (LOQ) has been established, as the assay was developed for sensitive detection rather than precise quantification at very low copy numbers. ddPCR runs were considered invalid in cases of failed droplet generation, insufficient accepted droplet count, positive droplets in the negative control, failed positive controls, or insufficient EMC7 amplification, indicating an inadequate sample quality. Samples with evidence of excessive genomic DNA contamination or poor cfDNA fragmentation quality were repeated or excluded from further analysis. The ddPCR assay is conducted at MDxCore, Department of Clinical Biochemistry, Copenhagen University Hospital—Rigshospitalet.

### 6.2. Data Management

Data will be stored on site in a secure REDCap web database [22,23]. The project leader and study investigators will be responsible for verifying the accuracy and completeness of the data.

### 6.3. Statistical Methods

The primary outcome, namely the sensitivity and specificity of ctHPV-DNA for detecting recurrence, will be calculated with 95% confidence intervals. Sensitivity will be determined among patients who develop recurrence, and specificity among patients who remain recurrence-free during follow-up. Positive predictive value will be determined among patients with positive ctHPV-DNA samples, and negative predictive value among patients with negative ctHPV-DNA results.

Only recurrence confirmed by a clinical and/or radiological examination will be regarded as recurrence. Histopathological confirmation will be included in the clinical verification if tissue sampling is feasible during the clinical examination.

The primary analyses will include all enrolled patients with a ctHPV-DNA baseline blood sample and at least one post-treatment blood sample, and follow-up assessment. Patients will be analyzed according to their clinical follow-up data, irrespective of the completeness of subsequent blood sampling or imaging, to minimize bias and reflect a real-world clinical practice.

Time-to-event outcomes, including time to recurrence detected by ctHPV-DNA and time to recurrence detected by clinical and/or radiological examination, will be analyzed using Kaplan–Meier survival curves. Median time to recurrence and corresponding 95% confidence intervals will be reported. Comparisons between ctHPV-DNA detection and standard surveillance will be performed using the log-rank test.

Healthcare utilization endpoints, including the number of PET/CT scans and Cancer Fast-Track referrals without evidence of recurrence, will be summarized using descriptive statistics (mean, median, standard deviation, and interquartile range). Between-group comparisons (e.g., patients with versus without recurrence) will be performed using *t*-tests for normally distributed variables or Mann–Whitney U tests for non-parametric distributions.

Analyses will use all available data, and, where appropriate, missing data will be handled using multiple imputation under the assumption that data are missing at random (MAR).

Sensitivity analyses will explore the impact of departures from the MAR assumption. For example, alternative imputation strategies may be applied, such as assuming that patients lost to follow-up either did or did not experience recurrence. These analyses will allow assessment of the robustness of estimates for sensitivity, specificity, time to recurrence, healthcare utilization, and patient-reported outcomes.

If the results from the sensitivity analyses are consistent with the primary analyses, confidence in the validity and generalizability of the findings will be strengthened. All statistical measures of uncertainty, including 95% confidence intervals and *p*-values, will be interpreted in the context of potential bias introduced by missing data.

Patient-reported quality of life (QoL), measured using the Danish version of the Fear of Cancer Recurrence Questionnaire (short form), will be evaluated as the change between follow-up visits. Each questionnaire item is rated on a five-point Likert scale (0–4) and summed into a total score. Total questionnaire scores will be reported, and within-patient changes will be assessed using the Wilcoxon signed-rank test. For repeated measurements over time, mixed-effects models using restricted maximum likelihood estimation will be employed, with patient ID as a random effect and time points as a fixed effect.

Exploratory analyses will include correlations between ctHPV-DNA levels and clinical outcomes, including recurrence, time to recurrence, and QoL scores. Spearman’s rank correlation coefficient will be used for non-parametric data. All tests will be two-sided, and a *p*-value < 0.05 will be considered statistically significant. All analyses will be conducted in R-studio.

## 7. Methods: Monitoring

### 7.1. Data Monitoring

A formal Data Monitoring Committee will not be established for this study. The study is a prospective, non-randomized diagnostic accuracy study involving serial blood sampling, without any therapeutic treatment or intervention expected to alter standard clinical care or pose more than minimal risk to participants. Therefore, no independent safety monitoring board is required.

An interim analysis will be conducted when all included patients have reached 1 year of follow-up. The interim analysis will include all study participants, and the results will be published in a peer-reviewed medical journal. As the study does not alter the standard treatment or follow-up program, no stopping guidelines have been defined.

### 7.2. Trial Monitoring

Oversight of study conduct and data integrity will be performed by the project leader and the study investigators. The study is conducted under institutional and sponsor oversight in accordance with all applicable ethical and regulatory requirements.

## 8. Ethics

### 8.1. Research Ethics Approval

This study has been approved by the Ethics Committee of the Capital Region of Denmark (H-23071576) and the Data Protection Office at Rigshospitalet (Forskningsjura; p-2023-15163).

### 8.2. Protocol Amendments

Any protocol amendment will be submitted to the Ethics Committee of the Capital Region of Denmark and must be approved before changes to the study are implemented.

### 8.3. Consent or Assent

Oral and written informed consent are obtained from all patients before inclusion in the study. Each patient will receive written information about the study together with the information pamphlets issued by the National Committee on Health Research Ethics describing participants’ legal rights. Thereafter, a member of the research group provides an oral presentation of the study and obtains written informed consent.

### 8.4. Confidentiality

All collected data will be kept confidential and stored in a secure REDCap database [22,23]. Each participant will be assigned a unique study identification number.

### 8.5. Ancillary and Post-Trial Care

No specific arrangements have been made for ancillary care or post-trial care. Participants will receive relevant treatment in accordance with standard healthcare practice and national DAHANCA guidelines. In the event of harm resulting from study participation, participants are covered by the national patient compensation scheme, as regulated by the Danish Act on the Right to Complain and Receive Compensation within the Health Services and administered by the Danish Patient Compensation.

## 9. Expected Results

This study uses the same ddPCR assay as our previously published retrospective study, which demonstrated a sensitivity of 97.2% for detecting ctHPV-DNA at the time of diagnosis in patients with HPV+ OPSCC and showed promising results for identifying residual disease or recurrence [12]. Based on these findings, we expect the assay to demonstrate high sensitivity and specificity for the prospective detection of recurrence.

## 10. Discussion

This study protocol outlines a prospective investigation of the sensitivity and specificity of ctHPV-DNA testing for detecting recurrence in patients with HPV+ OPSCC. The study will be population-based, with all eligible patients diagnosed with HPV+ OPSCC in Eastern Denmark invited to participate. The goal is to enable earlier and more accurate detection of recurrence in this patient population. If ctHPV-DNA can reliably identify patients with recurrent disease at an earlier stage, it may facilitate timely and earlier clinical intervention while reducing unnecessary diagnostic procedures and their associated risks and side effects. Furthermore, patients diagnosed and treated at an earlier stage typically undergo less extensive treatment, which is associated with fewer permanent side effects than treatment for patients diagnosed with advanced disease.

Additionally, patients in post-treatment follow-up programs often worry about recurrence, which can significantly impact their quality of life. Currently, detection of a recurrence relies on the presence of symptoms or visible tumor recurrence during clinical examination. Clinicians can therefore only rule out clinically evident recurrence. The presumed high sensitivity of ctHPV-DNA testing for detecting recurrence may therefore provide patients with a greater sense of reassurance during follow-up and also provide clinicians with a reliable tool for recurrence detection. The main concern with ctHPV-DNA testing is the occurrence of positive ctHPV-DNA blood samples without evidence of clinical recurrence. This uncertainty could understandably increase patients’ anxiety. However, by including the Danish *Fear of Recurrence Questionnaire*, we will be able to assess how test results influence patients’ fear of recurrence throughout the study.

The existing literature on ctHPV-DNA in HPV+ OPSCC is limited by relatively small study populations, short follow-up, or only sporadic sampling during follow-up, and frequently focuses exclusively on HPV16-positive OPSCC [24,25,26,27,28,29,30]. This protocol addresses these limitations by including a large study population followed prospectively and systematically, with ctHPV-DNA blood sampling at baseline and at every follow-up visit after treatment completion, including clinical action on positive results. In addition, this study investigates a wide panel of HPV genotypes.

## Data Availability

According to Danish data protection law, data from this study are not allowed to be made publicly available. Transfer of data requires permission from the Danish data protection authorities for the specific transfer. This can be applied for upon request to the corresponding author.

## Supplementary Materials

The following supporting information can be downloaded at: https://www.mdpi.com/article/10.3390/diagnostics16142169/s1, SPIRIT 2025 checklist. Reference [31] is cited in the Supplementary Materials.
